# Paraneoplastic encephalitis and delirium – a case report

**DOI:** 10.1192/j.eurpsy.2023.2003

**Published:** 2023-07-19

**Authors:** K. Jejcic, M. Stojanovic

**Affiliations:** 1 Dispensary for Psychohygiene, Health Center Maribor; 2 Department of Psychiatry, University Clinical Center Maribor, Maribor, Slovenia

## Abstract

**Introduction:**

Delirium is an important mental disorder, especially in intensive care units, which negatively affects the morbidity and mortality. Subjective clinical assessment of patients by non-psychiatric health professionals in intensive care units is insufficient to detect and measure delirium. Therefore, different scoring scales have been developed to assess delirium. A brief examination cannot entirely differentiate between a delirium, especially of organic origin, versus a psychotic break. Measurement scales for delirium are not routinely used. However, evidence shows that objective assessment of delirium contributes to its early detection in intensive care and the initiation of appropriate treatment.

**Objectives:**

To show the importance of using validated scales in delirium patients.

**Methods:**

A case report.

**Results:**

A 63-year-old male patient was admitted to our psychiatry ward after being evaluated by the local internal medicine specialist for confusion and suicidal ideation. He wrote a suicide note and had a positive family history for mental disorder (the brother has schizophrenia). During the initial mental state examination, the patient showed general disorientation, thought dissociation and defunct reality testing. A profound laboratory testing did not show any meaningful changes. A CT-scan was conducted that showed no pathologic alteration. Firstly, the patient was treated as a psychotic case, with haloperidol and diazepam parenterally. After no evident improvement of his mental state the Delirium Detection Scale (DDS) was used. Eliciting a highly positive result, the patient was re-evaluated as an (somatic) delirium. Therefore, a neurologist was consulted and a lumbar puncture performed. The cerebrospinal fluid (CSF) was indicative for a viral meningitis. So, the patient was admitted to the Infectious Disease Unit. As no treatment showed results an additional CSF panel for paraneoplastic antibodies. This came back very positive for AntiHu – a marker for small-cell lung carcinoma. Next day’s thoracic CT scan revealed a massive carcinoma with no proliferation. At last, the patient was transferred to the pneumo-oncological unit where he received pulse therapy with methylprednisolone. After which, his mental state recovered fully and the patient started chemotherapy.

**Conclusions:**

Delirium is a complex medical situation. It is an emergency which is classified in the ICD as a mental disorder. However, it demands medical and non-psychiatric therapy swiftly. Only, a swift and precise diagnosis can be a leading light here. The use of diagnostic scales should be encouraged as shown in this case report.

**Disclosure of Interest:**

None Declared

